# Improved urethral fluorescence during low rectal surgery: a new dye and a new method

**DOI:** 10.1007/s10151-018-1757-6

**Published:** 2018-02-19

**Authors:** T. G. Barnes, D. Volpi, C. Cunningham, B. Vojnovic, R. Hompes

**Affiliations:** 1 Nuffield Department of Surgical Sciences, John Radcliffe Hospital, University of Oxford, Level 6, Headley Way, Headington, Oxford, OX3 9DS UK; 2 Department of Colorectal Surgery, Oxford University Hospitals NHS Foundation Trust, Oxford, UK; 3 Department of Oncology, CR-UK/MRC Oxford Institute for Radiation Oncology, University of Oxford, Oxford, UK

**Keywords:** Colorectal surgery, Fluorescence, Laparoscopic surgery, TaTME, Urethral injury

## Abstract

**Background:**

The aim of this study was to demonstrate highlighting of the urethra during surgery through the use of two different methods: a new near-infrared fluorophore IRDye800BK, and indocyanine green (ICG) mixed with silicone.

**Methods:**

Male cadavers from the department of anatomy at the University of Oxford were used to visualise the urethra during near-infrared fluorescence excitation. To assess IRDye800BK, a perineal incision was utilised after infiltrating the urethra directly with an IRDye800BK solution mixed with Instillagel. ICG-silicone was assessed when the urethra was purposely exposed as part of a simulated transanal total mesorectal dissection. ICG was previously mixed with ethanol and silicone and left to set in a Foley catheter. Fluorescence was visualised using an in-house manufactured fluorescence-enabled laparoscopic system.

**Results:**

IRDye800BK demonstrated excellent penetration and visualisation of the urethra under fluorescence at an estimated tissue depth of 2 cm. An ICG-silicone catheter demonstrated excellent fluorescence without leaving any residual solution behind in the urethra after its removal.

**Conclusions:**

The newly described ICG-silicone method opens up the possibility of new technologies in this area of fluorescence guided surgery. IRDye800BK is a promising alternative to ICG in visualising the urethra using fluorescence imaging. Its greater depth of penetration may allow earlier detection of the urethra during surgery and prevent wrong plane surgery sooner.

**Electronic supplementary material:**

The online version of this article (10.1007/s10151-018-1757-6) contains supplementary material, which is available to authorized users.

## Introduction

Transanal total mesorectal excision (TaTME) has received much attention in the literature following the report on the first clinical case in 2010 [1]. TaTME aims to address the limitations of TME surgery, particularly for low rectal cancers, and to improve oncological outcomes [2]. TaTME can be a technically challenging procedure.

One of the main concerns with the ‘bottom-up’ part of TaTME is injury to the urethra [3] which has been documented in a number of cases [4]. Initial registry data reported urethral injury at a rate of 1% (5/489) [5] which is likely to be under-reported. Higher incidence rates have been reported in single centre cohort series, from 2% [6] to 6.7% [7].

Anatomical landmarks are key in preventing iatrogenic urethral injury. The anterior plane in male patients, particularly below the apex of the prostate, is challenging, and no clear landmarks exist. Fear of breaching the rectal wall or encroaching on an anteriorly located tumour can lead to surgeons dissecting in a more anterior plane than required, risking direct injury to the membranous (preprostatic) urethra [8]. Alternatively, dissection too far lateral can lead to prostatic mobilisation and dislocation of the prostate inferiorly while still attached to the rectal wall, exposing the membranous urethra. If not recognised, the prostate will appear to be part of the rectum and further anterior dissection will lead to urethral injury [9, 10].

One way to enhance visualisation of the urethra is by using fluorescence in the near-infrared (NIR) light spectrum. Fluorescence relies on particular molecules (fluorophores) that become ‘excited’ during exposure to light over a specific wavelength range followed by emission of light at longer wavelengths. A fluorescence-enabled system typically consists of a source to provide excitation light (e.g. by laser or filtered light) and an imaging system that blocks unwanted reflected light outside of the emission range of the fluorophore [11].

Urethral fluorescence using indocyanine green (ICG) has previously been described [6, 12]. ICG is a cyanine-based NIR fluorescent dye originally used for angiography in ophthalmology, as it allows increased tissue penetration depth to be achieved, superior to that associated with fluorescein.

We describe two different novel methods to help localise the urethra using NIR fluorescence with a new dye preclinical (IRDye800BK) and by means of an ICG-silicone mix.

## Materials and methods

Two male cadavers obtained from the department of anatomy at the University of Oxford were used to demonstrate both techniques. Both cadavers were utilised as part of a TaTME cadaveric workshop [13]. Consent for teaching and research was obtained from all patients donating their bodies to medical science. The study was approved by the department of anatomy ethics committee.

For perineal urethral visualisation, a vertical incision was made in the perineum with visualisation of the urethra under fluorescence attempted at different depths of dissection when exposing the urethra. A total of 2.5 mg of IRDye800BK (LI-COR Biosciences ^®^, Lincoln, Nebraska, USA) dye was mixed with 1 ml of water and with 10 ml of anaesthetic antiseptic lubricant (Instillagel ^®^, High Wycombe, Bucks, UK). The IRDye800BK solution was then infiltrated directly into the urethra via the urethral meatus prior to dissection following a procedure described previously [12].

**Fig. 1:**
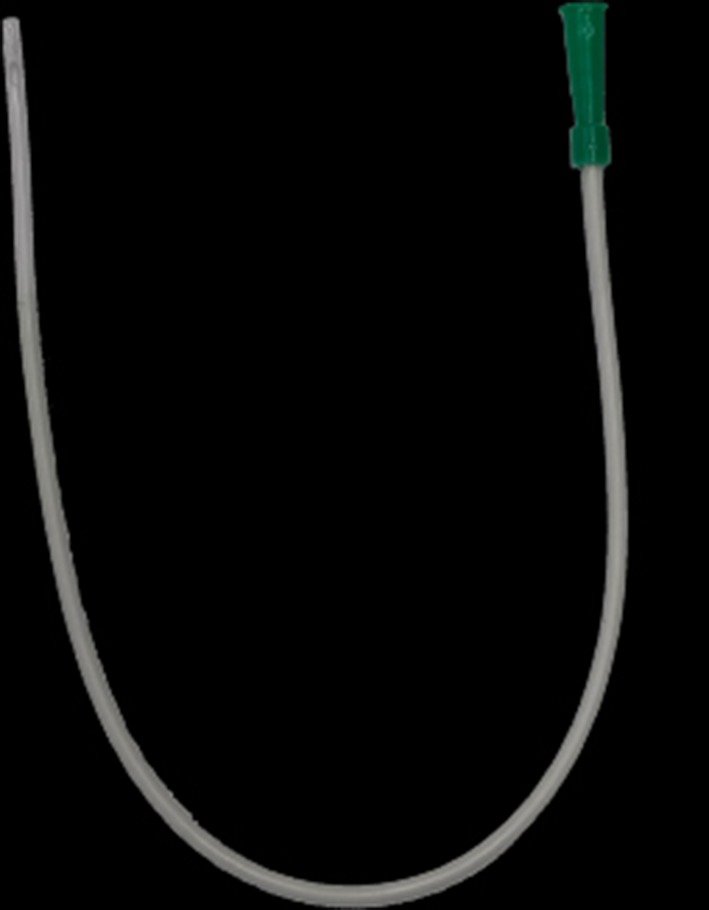
Image of a one-way urinary catheter filled with ICG-silicone mix

For transanal urethral visualisation (as part of TaTME), 2 mg of ICG (Diagnostic Green, Aschheim-Dornach, Germany) was dissolved in 1 ml of 100% ethanol and mixed with 10 ml of transparent silicone (RS Pro, Corby, Northants, UK). The ICG-silicone solution was infiltrated into a 10-Fr one-way Foley catheter and allowed to set for 1 week (Fig. 1). The catheter was placed in the urethra using a standard technique.

Visualisation of fluorescence was performed using an in-house manufactured fluorescence-enabled laparoscopic imaging system described previously by Volpi et al. [14]. Conventional white light illumination was combined with 785-nm laser light and delivered to a 10-mm rigid laparoscope (26003AGA, Karl Storz, Tuttlingen, Germany). The laparoscope working distance was set to ~ 50 mm, resulting in an excitation power density at the tissue surface of ~ 15 mW/cm^2^. A single colour camera was used for simultaneous detection of fluorescence emission (> 805 nm) and conventional white light reflectance. The camera exposure time was set to 40 ms to ensure real-time imaging. Ambient light was reduced to a minimum to limit unwanted background light. Images were displayed on an external monitor for visualisation and were recorded using a laptop. Signal-to-background ratios were assessed using ImageJ [15] software by drawing a region of interest around the urethra and measuring the brightness of the signal. The same size region of interest then measured background signal to devise the ratio.

## Results

### IRDye800BK for urethral fluorescence

**Fig. 2:**
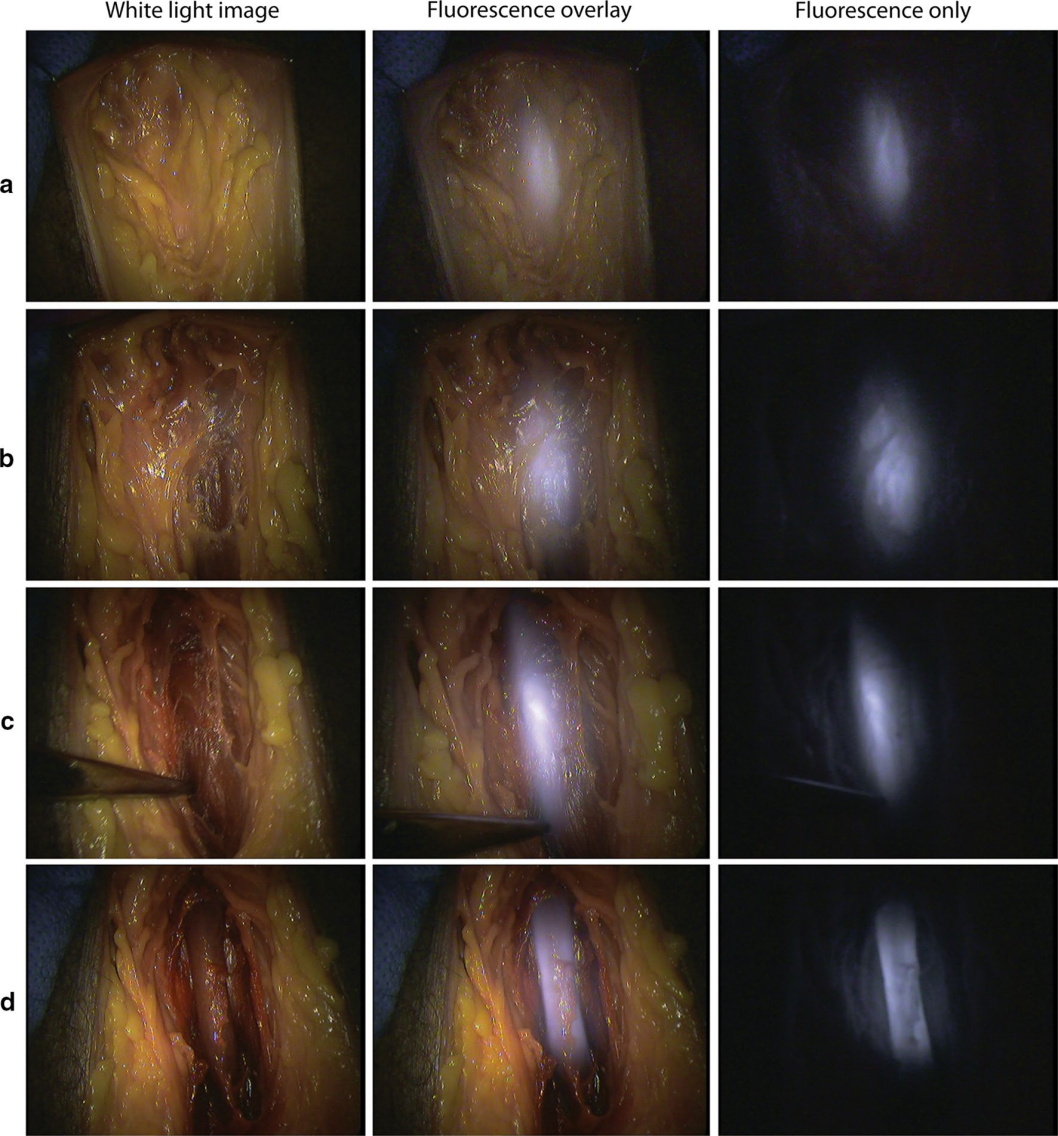
Demonstration of urethral fluorescence using IRDye800BK. From **a** to **d** there is an increasing depth of dissection. Row a demonstrates only an incision through epidermis and dermis with a clear fluorescent signal depicting the urethra at an estimated depth of 2 cm

After the perineal skin was incised, prior to dissecting through any fat overlying the urethra, an attempt was made to visualise fluorescence in the urethra. A clear, defined signal was observed in the location of the urethra with 2 cm of overlying fat. Deeper dissection was performed in order to expose the denuded urethral tube, clearly identifying the fluorescence located only within the urethra (Fig. 2). Signal-to-background ratios of each of the images are reported in Table 1.

**Table 1:** Fluorescence signal to background data in Fig. 2a–d

Image (from Fig. 2)	Signal	Background	Signal-to-background ratio
a	86.336	24.257	3.559220019
b	87.175	16.516	5.278215064
c	99.882	16.002	6.241844769
d	102.248	15.478	6.60602145

### ICG-silicone

**Fig. 3:**
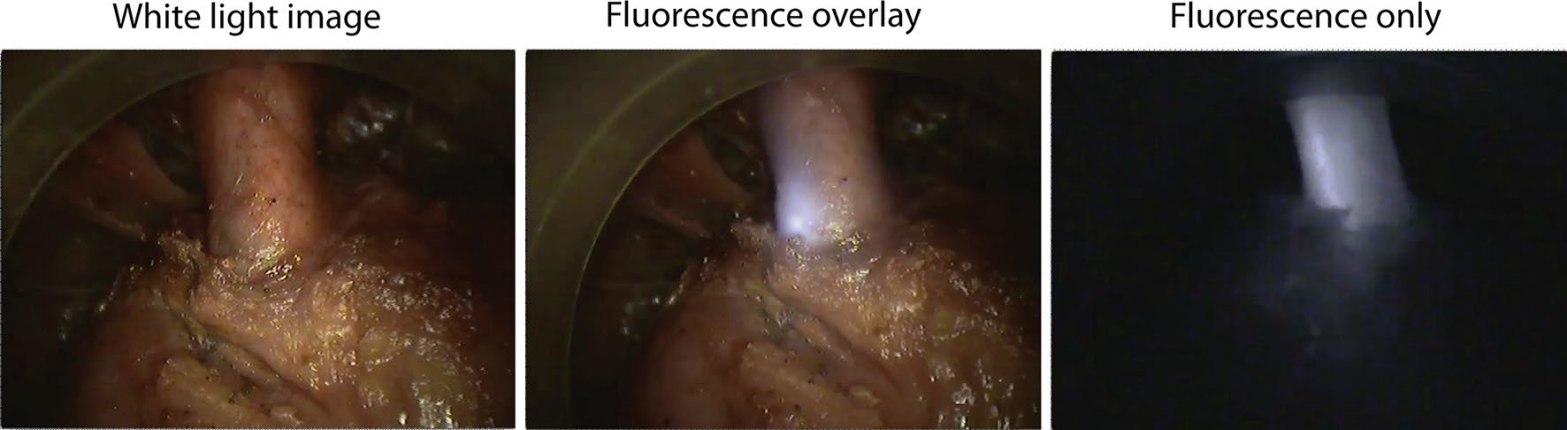
ICG-silicone catheter in the urethra during cadaveric TaTME dissection. Left image is white light only, middle image is fluorescence overlay, and right image is fluorescence only. Note that the three images were acquired in rapid succession, and therefore, a short time delay may be present

During the transanal part of TaTME dissection, in the anterior plane, the membranous urethra was exposed. After placement of the ICG-silicone catheter, fluorescence was observed clearly through the urethra (Fig. 3). Following removal of the silicone filled catheter, no residual fluorescence was observed indicating no leakage of ICG-silicone from the catheter (see supplementary video 1). Signal-to-background ratio of the denuded urethra was 5.75.

## Discussion

Our study outlines two new methods of urethral fluorescence with the use of a new near-infrared dye (IRDye800BK) mixed with Instillagel directly into the urethra and a new method of ICG mixed with silicone.

ICG has previously been shown to be useful in highlighting the urethra [8, 12], but, to the best of our knowledge, this is the first time its use with silicone has been described. Silicone is a well-known, medically safe material used in a wide variety of indwelling medical devices. Mixing silicone with dissolved ICG is a novel way of adapting the material for use in surgery and could lead to future developments such as fluorescent urinary catheters. However, ICG fluoresces in a dilute form. A drawback of ICG in this setting is that its fluorescence emission over time is reduced, suggesting that only limited storage times are possible. Alternatives to overcome this may include inorganic fluorophores such as lanthanides or quantum dots [16].

Following our previous method [12], the use of IRDye800BK appears to provide an excellent depth of penetration and brightness and could be a superior alternative to ICG fluorescence-based urethral localisation during low rectal surgery. Our previous study showed that with ICG a good tissue penetration (around 1 cm) was achieved with dissection in the perineum to expose the urethra [12]. The combination of IRDye800BK and an in-house manufactured fluorescence-enabled laparoscope with optical excitation source and imaging filters compatible for this fluorophore provided a higher depth of penetration when compared to our previously published results that used ICG and a commercial fluorescence system [12]. This allows potentially deeper and therefore earlier visualisation of the urethra by the surgeon using fluorescence and therefore wrong plane surgery can be detected quicker.

It is well known that NIR light penetrates through thicker tissue than white light [17]. The signal-to-background ratio if IRDye 800BK was higher both with a denuded urethra (6.61) and with covering tissue (6.24) when compared with ICG in silicone (5.75) in a denuded urethra. Whilst this suggests superiority of IRDye 800BK, further dosing evaluation would need to be conducted.

A further application would be to explore the use of IRDye 800BK dye mixed with silicone. Direct infiltration of a clinical dye would require a clinical trial which would come under the Medicines and Healthcare products Regulatory Authority (MHRA) or Food and Drug Administration (FDA) regulations for investigational medicinal products. This entails considerable cost and time to initiate. However, when mixed with silicone and placed in the patient, it is likely that it would be considered a device and therefore regulated under the device regulations, which are less burdensome [18], allowing in vivo assessment of this new dye. There are currently no published data of the use of IRDye 800BK in humans although our institution will be testing intravenous use of IRDye 800BK during colorectal surgery [19].

## Conclusions

The newly described ICG-silicone method opens up the possibility of new technologies in fluorescence guided surgery. IRDye800BK is a promising alternative to ICG in visualising the urethra. Its greater depth of penetration may allow earlier detection of the urethra during surgery and thus prevent wrong plane surgery sooner.

## Electronic supplementary material

Below is the link to the electronic supplementary material.

Supplementary Video 1 ICG-silicone catheter in the urethra during cadaveric TaTME dissection. (MP4 72956 kb)
